# Affinity Propagation Clustering of Measurements for Multiple Extended Target Tracking

**DOI:** 10.3390/s150922646

**Published:** 2015-09-08

**Authors:** Tao Zhang, Renbiao Wu

**Affiliations:** 1 School of Electronic Information Engineering, Tianjin University, Tianjin 300072, China; 2 Tianjin Key Laboratory for Advanced Signal Processing, Civil Aviation University of China, Tianjin 300300, China; E-Mail: tzhang@tju.edu.cn

**Keywords:** multiple extended target tracking, measurement partitioning, affinity propagation clustering, probability hypothesis density filter, elliptical gating

## Abstract

More measurements are generated by the target per observation interval, when the target is detected by a high resolution sensor, or there are more measurement sources on the target surface. Such a target is referred to as an extended target. The probability hypothesis density filter is considered an efficient method for tracking multiple extended targets. However, the crucial problem of how to accurately and effectively partition the measurements of multiple extended targets remains unsolved. In this paper, affinity propagation clustering is introduced into measurement partitioning for extended target tracking, and the elliptical gating technique is used to remove the clutter measurements, which makes the affinity propagation clustering capable of partitioning the measurement in a densely cluttered environment with high accuracy. The Gaussian mixture probability hypothesis density filter is implemented for multiple extended target tracking. Numerical results are presented to demonstrate the performance of the proposed algorithm, which provides improved performance, while obviously reducing the computational complexity.

## 1. Introduction

Multi-target tracking involves estimating the current state (e.g., position, speed, *etc*.) of targets using the measurements from multiple targets. Many approaches have been proposed for solving this problem [1,2]. In most target tracking cases, it is assumed that at most one measurement is produced by the target per scan, but this is not true in some cases, e.g., more measurements per scan are potentially generated by the target when it is detected by a high resolution sensor, or there are more than one measurement source on the target surface. This is denoted as an extended target.

The Random Finite Sets (RFS) theory has been proposed to solve the problem of multi-target tracking [3]. Mahler proposed the probability hypothesis density (PHD) filter [4], which is a first order moment approximation in the RFS framework, and has been shown to be a computational alternation. Then Mahler presented an extension of the PHD filter to tackle the problem of multiple extended target tracking, which is called the extended target PHD (ET-PHD) filter [5]. Granstrom proposed an application for the ET-PHD filter with the linear Gaussian assumption [6,7], which is referred to as the extended target GM-PHD (ET-GM-PHD) filter.

The PHD filter for multiple extended target tracking has drawn considerable attention [8,9]. However, the problem of how to partition the measurements of multiple extended targets is a crucial problem. In a multiple extended targets scene, it is very difficult to obtain the relationship between measurements and targets, and divide measurements from one target into the same partition. To address the difficulty of measurement partitioning for multiple extended target tracking, Granstrom proposed a partitioning method based on the distances between measurements [6,7], Zhang proposed an algorithm using the fuzzy ART model [10], and Yang used a spectral clustering algorithm for partitioning [11]. However, the problem of how to accurately and effectively partition the measurements of the extended target with clutter remains unsolved. A novel measurement partitioning algorithm based on affinity propagation clustering for multiple extended target tracking is proposed in this paper. Firstly, the elliptical gating technique is introduced to remove the clutter measurements, and then the affinity propagation clustering algorithm is used to partition the measurements, and finally the ET-GM-PHD filter is implemented for tracking multiple extended targets. Numerical results are provided to demonstrate the effectiveness and performance of the proposed algorithm, which obviously reduces the computational complexity, while obtaining improved performance.

The reminder of this paper is organized as follows: in Section 2, the measurement partitioning problem and the conventional methods are described. In Section 3, the ET-GM-PHD filter is summarized, and then the novel measurement partitioning algorithm for multiple extended target tracking based on affinity propagation are proposed in Section 4. Numerical simulations are presented in Section 5. The conclusions are drawn in Section 6.

## 2. Problem Statement and State-of-the-Art Methods

### 2.1. Measurement Partitioning

Measurement partitioning is a matter of importance for extended target tracking, since the target potentially generates more measurements per scan. The purpose of measurement partitioning is to collect measurements from one target into the same partition. In a dense target and clutter environment, it is hard to partition the measurements efficiently and accurately. In order to illustrate the process of measurement partitioning, let us assume there are three measurements in the measurement set $Z=\{z_{1},z_{2},z_{3}\}$. The measurement set can be divided into five potential partitions:

$$\begin{array}{l} p_{1}\begin{array}{c} : \end{array}\;W_{1}^{1}=\{z_{1},z_{2},z_{3}\}\\ p_{2}\begin{array}{c} : \end{array}\;W_{1}^{2}=\{z_{1}\}\begin{array}{cc} , & W_{2}^{2}\{z_{2}\} \end{array}\begin{array}{cc} , & W_{3}^{2}=\{z_{3}\} \end{array}\\ p_{3}\begin{array}{c} : \end{array}\;W_{1}^{3}=\{z_{1},z_{2}\}\begin{array}{cc} , & W_{2}^{3}=\{z_{3}\} \end{array}\\ p_{4}\begin{array}{c} : \end{array}\;W_{1}^{4}=\{z_{1},z_{3}\}\begin{array}{cc} , & W_{2}^{4}=\{z_{2}\} \end{array}\\ p_{5}\begin{array}{c} : \end{array}\;W_{1}^{5}=\{z_{1}\}\begin{array}{cc} , & W_{2}^{5}=\{z_{2},z_{3}\} \end{array} \end{array}$$

where $W_{i}^{j}$ denotes the *i*-th cell of the partition j.

All the potential partitions are required to update the current states of targets in the original ET-PHD filter. As the cardinality of the measurement set grows, the number of potential partitions grows very quickly. In a dense target and clutter environment, this makes the ET-PHD filter computationally intractable.

### 2.2. State-of-the-Art Methods of Partitioning

As a crucial problem, measurement partitioning has drawn considerable attention in multiple extended target tracking studies. In [6] Granstrom proposed a distance-based partitioning method. Given a set Z of measurements and a distance threshold set ${\left\{d_{i}\right\}}_{i=1}^{N_{d}}$ with $d_{i}<d_{i+1}$, and the distance is the Mahalanobis distance between two measurements. For each threshold $d_{i}$, the measurement set can be divided into a unique partition, in which the distance between all pairs of measurements in the same cell are less than $d_{i+1}$. Therefore, $N_{d}$ different distance thresholds can generate $N_{d}$ measurement partitions. Note that as the $d_{i}$ increases, each partition contains fewer cells, while the cells contain more measurements. Because the Mahalanobis distance between the measurements is $\chi^{2}$ distribution, using the inverse $\chi^{2}$ distribution, a distance threshold $\delta_{P_{G}}$ can be computed for a given probability $P_{G}$. However, each distance threshold generates a unique measurement partition, so it needs a suitable threshold set to generate lots of potential partitions including the correct partition. The simulation results show when set distance thresholds $\delta_{P_{L}}<d<\delta_{P_{U}}$, with $P_{L}=0.3$ and $P_{U}=0.8$, extended target tracking obtains good results.

In [10] Zhang proposed an algorithm using the fuzzy Adaptive Resonance Theory (ART) model to solve the difficulty in measurement partitioning. Different vigilance values can generate alternative partitions of the measurements in ART, thus $N_{V}$ alternative partitions are generated by $N_{V}$ different vigilance values ${\left\{\rho_{l}\right\}}_{l=1}^{N_{V}}$, where $\rho_{l+1}=\rho_{l}+\Delta$, and Δ is a vigilance gain. Large gain generates fewer alternative partitions, whereas small gain generates more alternative partitions. A bad choice of gain will generate extra partitions and computation time.

In [11] Yang proposed the spectral clustering algorithm for partitioning. Firstly, a similarity matrix is built by calculating the Euclidean distance between each pair of measurements, then an eigen matrix is constructed by using the eigenvectors corresponding to the K largest eigenvalues, finally, K-means++ clustering is used to partition the measurements. K is the cluster number with $K\in (K_{L},K_{U})$, the minimum cluster number is $K_{L}=\left[\frac{N_{G}}{2\beta }\right]$ and the maximum cluster number is $K_{U}=\left|M\right|$, where $N_{G}$ is the number of measurements, and β denotes the measurement rate conforming to the Poisson distribution. |M| is an integer between the number of targets and measurements, however, it is very difficult to accurately estimate the number of targets before the tracking process.

### 2.3. Existing Problems of State-of-the-Art Methods

Although the measurement set can be partitioned by the state-of-the-art methods, they are not efficient and accurate enough. In the Distance Partitioning algorithm, each distance threshold only can produce a unique partition. In order to get the correct partition, lots of distance thresholds are needed to generate enough partitions, and the distance thresholds are empirical values. As the number of targets increases, this makes the extended target tracking process computationally intractable. Although the fuzzy ART model and spectral clustering method reduce the computational burden, the partitioning results would become rather bad in a cluttered dense scene, and the results are also dependent on the selection of the cluster parameters. In order to tackle the problem of the efficiency and accuracy of partitioning method, a novel measurement partitioning algorithm is proposed based on affinity propagation clustering. Compared with the state-of-the-art partitioning methods, affinity propagation clustering retrieves the number of partition iteratively, so we do not have to set clustering parameters, and it always achieves better partitioning results with less computational cost.

## 3. ET-GM-PHD Filter

Mahler expanded the PHD filter for extended target tracking in [5], which is called ET-PHD filter. Granstrom introduced the Gaussian mixture model to the ET-PHD filter, and a proximate solution is proposed, which is referred to as the ET-GM-PHD filter. The ET-GM-PHD filter is summarized as follows, and detailed descriptions can be found in [5,12,13,14,15].

More measurements are potentially generated by the extended target per scan. Assuming the number of extended measurements is a Poisson distribution, the probability of at least one measurement generated by the *i*-th target generated is given as:

(1) $$1-e^{-\gamma (x_{k}^{\left(i\right)})}$$

where $\gamma (x_{k}^{\left(i\right)})$ denotes the expected number of measurements, which are generated by the *i*-th extended target at time step k, and $e^{-\gamma (x_{k}^{\left(i\right)})}$ is the probability that the *i*-th target will not generate extended measurements.

The *i*-th target is detected with the probability $P_{D}(x_{k}^{\left(i\right)})$, and the effective probability of detection is then given as:

(2) $$\left(1-e^{-\gamma (x_{k}^{\left(i\right)})})P_{D}(x_{k}^{\left(i\right)}\right)$$

The target state $x_{k}^{\left(i\right)}$ is modeled using the linear Gaussian dynamics model, and the measurements are also assumed to follow the linear Gaussian model. The extended measurements are modeled as the spatial probability distribution [16], and the number of extended measurements is a Poisson distribution. The clutter has a Poisson distribution and its intensity can be described as $\lambda_{k}c_{k}(z_{k})$, where $\lambda_{k}$ and $c_{k}(z_{k})$ are the mean number and the space distribution of the clutter measurements, respectively.

Assuming that $\upsilon_{k-1|k-1}(x)$ is the intensity of extended targets at time step k−1, the prediction equations of the ET-GM-PHD filter are given as:

(3) $$\upsilon_{k|k-1}(x)=\upsilon_{S,k|k-1}(x)+\gamma_{k}(x)$$

where $\upsilon_{S,k|k-1}(x)$ is the predicted intensity of the survived targets, and $\gamma_{k}(x)$ is the spontaneous birth target with RFS form.

The update formulas for the ET-GM-PHD filter are given by:

(4) $$\upsilon_{k|k}(x)=\upsilon_{k|k}^{ND}(x)+\sum_{p∠Z_{k}}\sum_{W\in p}\upsilon_{k|k}^{D}(x,W)$$

where the Gaussian mixture $\upsilon_{k|k}^{ND}(x)$ tackles the no detection cases, and $\upsilon_{k|k}^{D}(x,W)$ tackles the detected target cases. p denotes a potential partition of the measurement set $Z_{k}$, W denotes the cell of the partition p.

## 4. Affinity Propagation Clustering of Measurement Set

### 4.1. Clutter Removal

**Figure 1:**
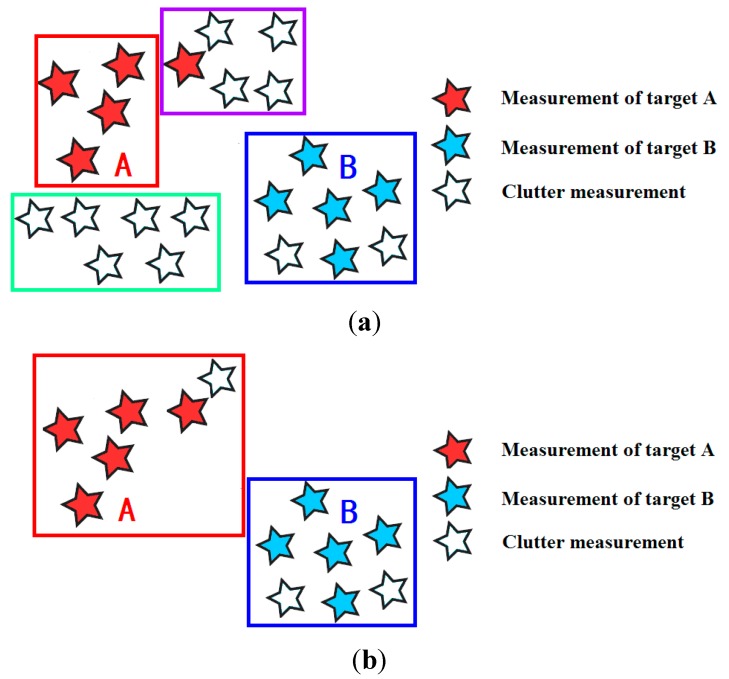
Influence of clutter measurements in partitioning (**a**) Partitioning before clutter removal (**b**) Partitioning after clutter removal.

In the multiple extended targets environment, the sensor not only receives measurements which are generated by the targets, but also receives a set of clutter measurements, which are not generated by any target. The clutter is modeled as a Poisson distribution with intensity $\lambda_{k}c_{k}(z_{k})$, where $c_{k}(z_{k})$ is the space distribution, and it is assumed as a uniform distribution over the surveillance region. The discrete distributed clutter measurements are mixed with the target-generated measurements. The efficiency and accuracy of partitioning are sensitive to the clutter, as dense clutter leads to a wrong partitioning result, and the corresponding sketch is shown in Figure 1a. To solve this problem, the elliptical gating technique as applied in the traditional tracking algorithm is introduced to remove partial clutter measurements. The sketch of clustering after measurement gating is shown in Figure 1b.

The elliptical gating defined by a validation region on the basis of the set of predicted measurements is used to remove clutter measurements. The residual error vector is defined as:

(5) $$\varepsilon^{\left(ji\right)}=z_{k}^{\left(i\right)}-H_{k}m_{k|k-1}^{\left(j\right)}$$

where $z_{k}^{\left(i\right)}$ is *i*-th measurement at time step k, and $H_{k}m_{k|k-1}^{\left(j\right)}$ denotes the *j*-th predicted measurement.

The covariance matrix of residual error is:

(6) $$S_{k}^{\left(j\right)}=H_{k}P_{k}^{\left(j\right)}H_{k}^{T}+R_{k}$$

where $H_{k}$ is the measurement matrix, $P_{k}^{\left(j\right)}$ is the predicted covariance matrix of x, and $R_{k}$ is the covariance matrix of measurement noise.

In the case of linear Gaussian system, the elliptical gate is the maximum likelihood gate [17], and it can be defined by:

(7) $${\left(\varepsilon^{\left(ji\right)}\right)}^{T}{\left(S_{k}^{\left(j\right)}\right)}^{-1}\varepsilon^{\left(ji\right)}\leq T_{g}$$

If $P_{g}$ is the probability of a target-generated measurement falling in the validation region, the 2-dimensional validation threshold $T_{g}=-2\ln(1-P_{g})$ is used to eliminate the clutter measurements in $Z_{k}$, and then the new measurement set $Z_{k,T}$ is obtained. In $Z_{k,T}$, most of the measurements are target-generated measurements. Only a few clutter measurements which are close to the target-generated measurements are not removed by the elliptical gate, but the measurement partitioning is not seriously restricted by these clutter measurements (see Figure 1b).

### 4.2. Affinity Propagation Clustering for Measurement Partitioning

Affinity propagation is a clustering algorithm, messages between data points are exchanged to find a subset of exemplar points that best describe the date points [18], a set of similarities between pairs of data points are given, and the goal of the algorithm is to minimize the overall sum of similarities between data points and their exemplars.

Let the measurements in $Z_{k,T}$ being the data points, and then we define the negative squared Euclidean distances between each pair of measurements as the similarities, given by:

(8) $$s(i,j)=-{\left\|z_{k}^{\left(i\right)},z_{k}^{\left(j\right)}\right\|}^{2}$$

where $z_{k}$ is the measurement in the validation region at time step k, and $z_{k}\in Z_{k,T}$.

The similarity matrix C of measurement set $Z_{k,T}=\left\{z_{1},\cdots ,z_{N_{Z}}\right\}$ is:

(9) $$C=\left\{\begin{array}{ccc} s(1,1) & \cdots & s(1,N_{Z})\\ \vdots & \ddots & \vdots \\ s(N_{Z},1) & \cdots & s(N_{Z},N_{Z}) \end{array}\right\}$$

where $N_{Z}$ is the number of the measurements in $Z_{k,T}$.

All the measurements have the same opportunity to be chosen as an exemplar measurement in the affinity propagation clustering algorithm, that is, it does not have to set the initial cluster centers, so the preference parameters ρ of any measurements are set to the same value.

The affinity propagation algorithm exchanges two messages between measurements. r(i,j) is referred to as “responsibility”, which is the message sent from measurement i to the candidate exemplar measurement j, and it denotes how well-suited measurement j is to be the exemplar for measurement i. a(i,j) is referred to as “availability”, which is the message sent from exemplar measurement j to measurement i, and it denotes how appropriate it would be for measurement i to serve as the exemplar of measurement j. The update formulas for r(i,j) and a(i,j) are given by:

(10) $$r(i,j)\leftarrow s(i,j)-\underset{j^{\prime }s.t.j^{\prime }\neq j}{\max}\left\{a(i,j^{\prime })+s(i,j^{\prime })\right\}$$

(11) $$a(i,j)\leftarrow \min\left\{0,r(j,j)+\sum_{i^{\prime }s.t.i^{\prime }\neq j}\max\left\{0,r(i^{\prime },j)\right\}\right\}$$

(12) $$a(j,j)\leftarrow \sum_{i^{\prime }s.t.i^{\prime }\neq j}\max\left\{0,r(i^{\prime },j)\right\}$$

According to the above-mentioned procedure, the messages exchanged between measurements are updated until an appropriately set of exemplars and partitions.

The proposed algorithm of affinity propagation clustering for extended target tracking using ET-GM-PHD filter can be summarized as following steps:

(1) Assuming that $\upsilon_{k-1|k-1}(x)$ is the intensity in RFS form of extended targets at time step k−1;
(2) The prediction of the extended target intensity $\upsilon_{k|k-1}(x)$ is calculated by Equation (3);
(3) Remove the clutter measurements:
  Given a threshold $T_{g}=-2\ln(1-P_{g})$, an elliptical region is defined with linear Gaussian assumption as follows:
  (13) $$\Omega (k,T_{g})=\{z_{k}:d_{k}={\left[z_{k}-Hm_{k|k-1}\right]}^{T}S_{k}^{-1}[z_{k}-Hm_{k|k-1}]\leq T_{g}\}$$
  where $\Omega (k,T_{g})$ denotes the validation region at time step k.
  The meaurements falling in the elliptical region can be obtained by:
  (14) $$Z_{k,T}=\{z_{k}\in Z_{k}|d_{k}\leq T_{g}\}$$
  The measurements out of the validation region can be removed as the clutter measurements;
(4) Measurements partitioning:
  Calculate the similarity matrix C using the negative Euclidean distance between each pair of the measurements in $Z_{k,T}$;
  All the target-generated measurements have the same opportunity to be chosen as an exemplar. In our study, the preferernce parameters are set to the mean value of the similarities, given by:
  (15) ρ=mean{s(i,j)}
  All the resonsibilities r and availabilities a are set to 0 initially, and then the “responsibility” r(i,j) and “availability” a(i,j) are updated iteratively, until they reach a specified value using Equations (10)–(12). The affinity propagation clustering retrieves the number of partitions iteratively, and then the optimal measurement partition $P_{k}$ is obtained without setting the parameters of the number of clusters;
(5) The predicted intensity $\upsilon_{k|k-1}(x)$ can be updated to $\upsilon_{k|k}(x)$ using the new measurement partition $P_{k}$ and Equations (3) and (4). Because partial clutter measurements are removed by the elliptical gating, the clutter intensity $\lambda_{k}c_{k}(z_{k})$ should be revised. The non-removed clutter in the validation region is also modeled as Poission distribution with $\lambda_{k}c_{k}(z_{k})=\lambda_{k}/V_{k}$, where $V_{k}$ is the volume of the validation region;
(6) The Gaussian component merging and pruning process are similar to the GM-PHD filter for standard targets tracking described in [13], and the state estimation of the extended targets also involves the estimation of target number and the extracting of the Gaussian mixture components with the highest weights from the posterior intensity as the state estimates.

### 4.3. Computational Complexity Analyses

In order to analysis the computational complexity of the partitioning algorithm, we define $N_{k}$ as the number of measurements at time step k. Before inputting to the affinity propagation clustering, creating the similarity matrix requires $N_{k}\times (N_{k}-1)$ operators, and then in the clustering algorithm, the message passing requires $N_{k}^{2}\log N_{k}$ operators. Because only the measurements located in the validation region defined by the elliptical gating are used for extended target tracking, the number of measurements input to the clustering algorithm is much less than $N_{k}$. Therefore, the worst case complexity of proposed partitioning is approximated as $O(N_{k}^{2})$.

For the distance partitioning method, in order to obtain the correct partition, lots of partitions are generated, so its complexity is much greater than $O(N_{k}^{4})$ [19].

For the ART partitioning method, the computational complexity involves creating the normalized measurement vectors, computing a category choice, and updating the weight vectors. The worst case complexity is approximated as $O(N_{k}^{2})$ [10]. However, in ART partitioning, the vigilance gain is an empirical value, and a bad choice of the gain will generate more extra partitions with more additional computation time.

For the spectral clustering partitioning method, the computational complexity involves creating the similarity matrix, calculating the Laplacian matrix, normalizing the similarity matrix and a K-means clustering. The worst case complexity is approximated as $O(N_{k}^{3})$.

## 5. Numerical Simulations

In order to present the performance improvements achieved with the proposed algorithm, we consider the case in which four targets with crossing a certain region of the [−100,100] × [−100,100] plane. The sampling period is T=1 s. Four targets are within the surveillance region in 1~40 s, 1~40 s, 10~35 s, 15~30 s, respectively. This means that two targets are always in the surveillance region, and the other two targets enter at 10 s, 15 s, and leave at 35 s, 30 s, respectively.

**Figure 2:**
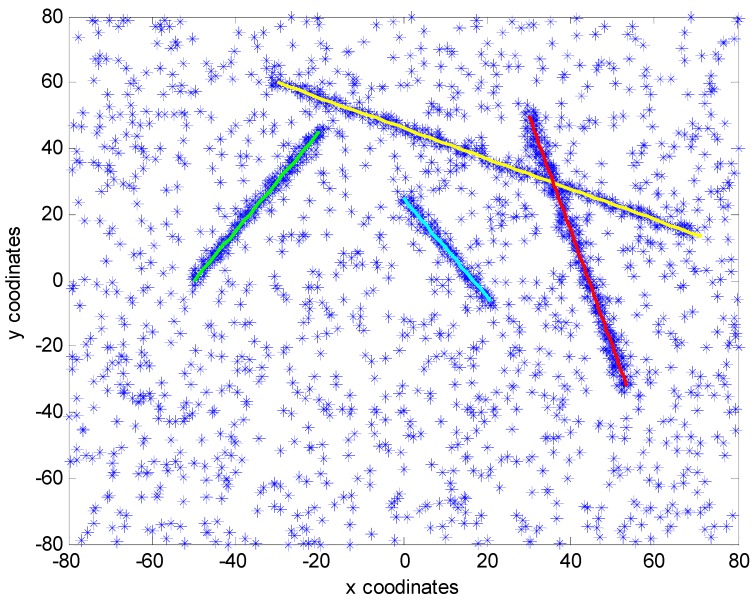
True trajectories and measurements.

The dynamics model and measurement model are described as linear Gaussian models, the probability of target survival $p_{S,k}$ is 0.99 and the probability of target detection $P_{D}$ is 0.9. The clutter is modeled as a Poisson RFS with the mean λ=50 and uniform density over the measurement space. The number of extended measurements is a Poisson distribution with the mean γ=10. Figure 2 shows the simulated scenario with true target trajectories (solid line) together with measurements (star) generated by the targets and clutters of 40 scans.

The RFS of spontaneous birth target is the Poisson distribution with intensity $\gamma_{k}(x)=\sum_{i=1}^{4}0.1N(x;m_{\gamma }^{i},P_{\gamma }^{i})$, where $m_{\gamma }^{1}={\left[-30,0,60,0\right]}^{T}$, $m_{\gamma }^{2}={\left[30,0,50,0\right]}^{T}$, $m_{\gamma }^{3}={\left[-50,0,0,0\right]}^{T}$, $m_{\gamma }^{4}={\left[0,0,25,0\right]}^{T}$, and $P_{\gamma }={\left[5,1,5,1\right]}^{T}$.

The elliptical gating is equipped with $P_{g}=0.95$, which ensures target-generated measurements can fall in the validation region rather than clutter measurements. The pruning threshold T is $10^{-5}$, the merging threshold U is 4, and the maximum of Gaussian components $J_{\max}$ is 200. The numerical simulations are implemented on a computer equipped with an Intel Core2 Quad 2.66 GHz CPU and 4 G RAM.

**Figure 3:**
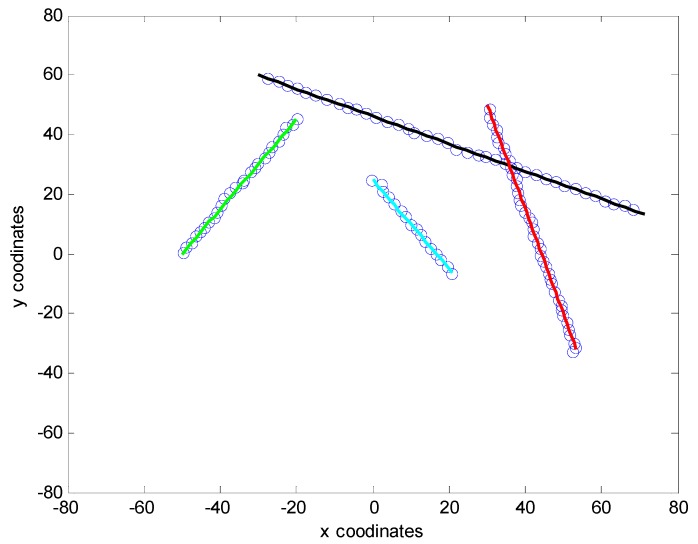
True trajectories and estimates using the proposed algorithm.

Figure 3 shows the ET-GM-PHD filter with the proposed affinity propagation clustering partitioning algorithm provides an accurate extended target tracking performance. The proposed filter not only successfully detects and tracks four extended targets, but also accurately detects the spontaneous birth and disappearance of targets.

In order to evaluate the performance of the proposed algorithm, 100 Monte Carlo numerical simulations are performed and two metrics are used, one is the estimate of the target number, the other is the optimal sub-pattern assignment (OPSA) distance [20].

**Figure 4:**
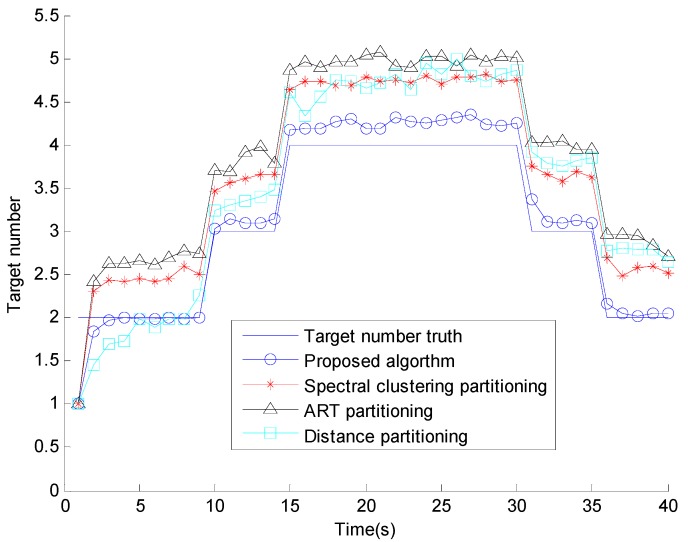
Target number estimates.

**Figure 5:**
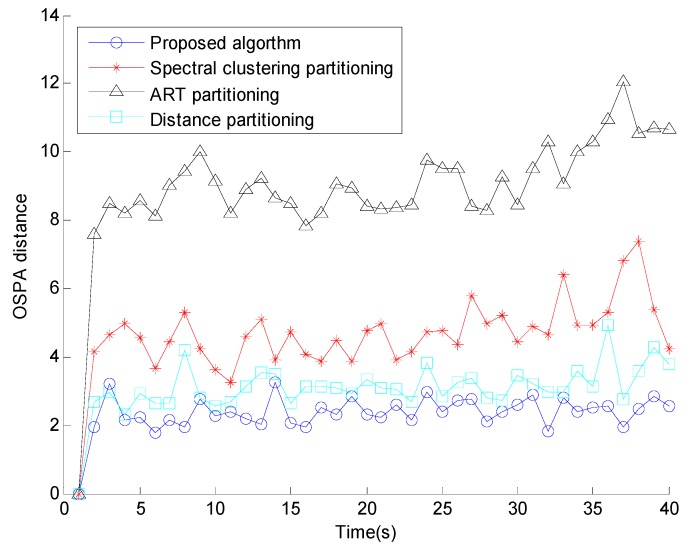
OSPA distance.

Figure 4 shows the number of estimates of extended targets of the four different methods, where it is clear that the proposed method obviously outperforms the others every time. The proposed algorithm obtains the most accurate number of estimates and it is the most similar one to the truth of the four methods. Figure 5 shows the OSPA distance, compared with the conventional methods, the proposed algorithm has the smallest OSPA distance, this is because that the performance of conventional partitioning algorithms are dependent on the selection of the cluster parameters and sensitive to the clutter measurements, but the proposed affinity propagation partitioning algorithm removes the clutter measurements by the elliptical gate, all the target-generated measurements have the same opportunities to be chosen as a cluster center, and retrieves the number of partition iteratively.

**Figure 6:**
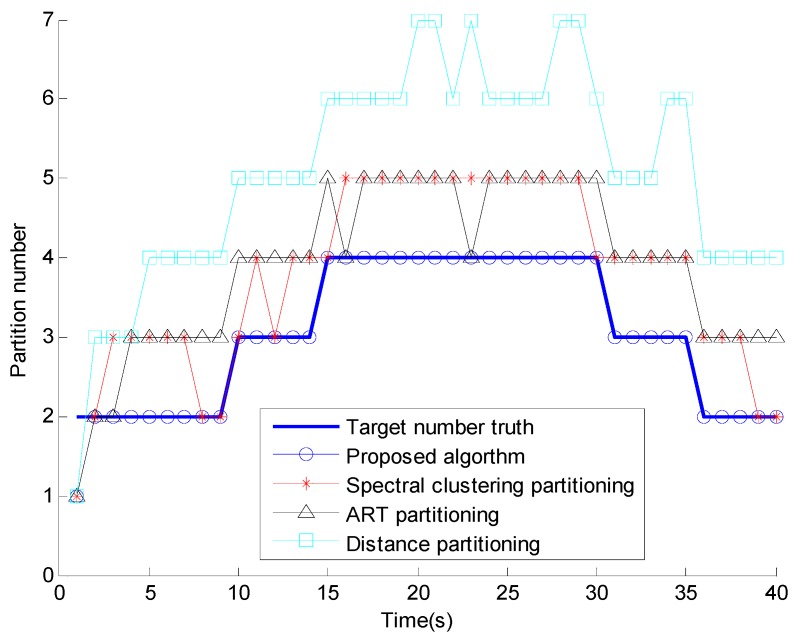
Partition number.

**Figure 7:**
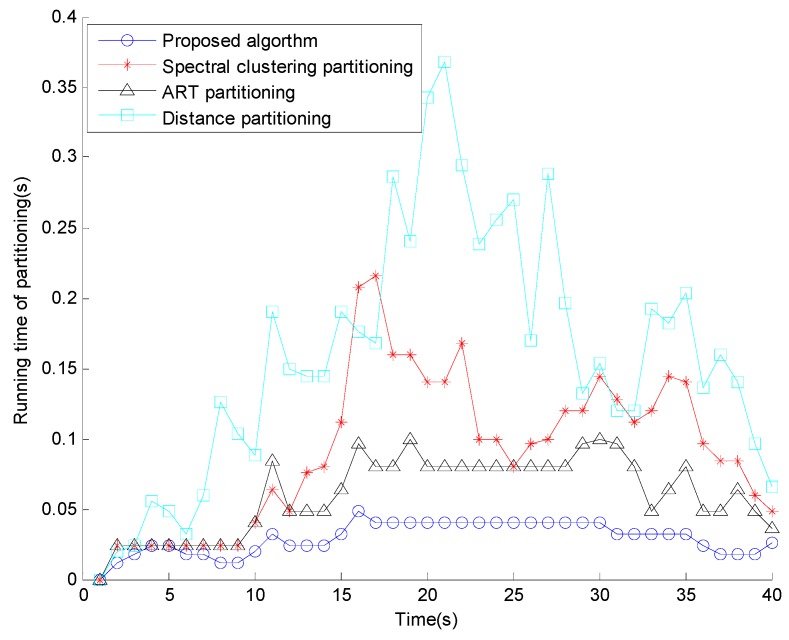
Running time of partitioning.

Figure 6 shows the comparison of the partition number and Figure 7 shows the running time comparison. The proposed algorithm has the most similar partition number to the truth and the computational cost is much smaller than that of the three conventional methods. The reason is that most of the clutter measurements are removed by the elliptical gating and the affinity propagation clustering retrieves the number of partition iteratively, while presenting outstanding computational complexity.

## 6. Conclusions

The affinity propagation clustering is introduced into measurement partitioning for extended target tracking for the first time, and the elliptical gating technique is used to remove the clutter measurements, which makes the affinity propagation clustering being capable of partitioning the measurement in a densely cluttered environment with high accuracy. The numerical results show a considerable performance improvement of the proposed algorithm both in the state estimate accuracy and the computational cost compared to the conventional methods.
